# Goals in motion: exploring goal setting among adults living with HIV who participated in an online community-based exercise intervention

**DOI:** 10.3389/fresc.2025.1644139

**Published:** 2025-07-31

**Authors:** Tai-Te Su, Soo Chan Carusone, Kiera McDuff, Francisco Ibáñez-Carrasco, Ada Tang, Ahmed M. Bayoumi, Mona Loutfy, Lisa Avery, George Da Silva, Annamaria Furlan, Helen Trent, Ivan Ilic, Zoran Pandovski, Mehdi Zobeiry, Puja Ahluwalia, Katrina Krizmancic, Tizneem Jiancaro, Brittany Torres, Patricia Solomon, Kelly K. O'Brien

**Affiliations:** ^1^ Department of Physical Therapy, Temerty Faculty of Medicine, University of Toronto, Toronto, ON, Canada; ^2^ McMaster Collaborative for Health and Aging, McMaster University, Hamilton, ON, Canada; ^3^ Dalla Lana School of Public Health, University of Toronto, Toronto, ON, Canada; ^4^ School of Rehabilitation Science, Faculty of Health Sciences, McMaster University, Hamilton, ON, Canada; ^5^ Institute of Health Policy, Management and Evaluation, Dalla Lana School of Public Health, University of Toronto, Toronto, ON, Canada; ^6^ MAP Centre for Urban Health Solutions, Li Ka Shing Knowledge Institute, St. Michael’s Hospital, Toronto, ON, Canada; ^7^ Department of Medicine, Temerty Faculty of Medicine, University of Toronto, Toronto, ON, Canada; ^8^ Division of General Internal Medicine, St. Michael’s Hospital, Toronto, ON, Canada; ^9^ Maple Leaf Medical Clinic, Toronto, ON, Canada; ^10^ Women’s College Research Institute, Women’s College Hospital, Toronto, ON, Canada; ^11^ Biostatistics Department, Princess Margaret Cancer Centre, University Health Network, Toronto, ON, Canada; ^12^ Realize, Toronto, ON, Canada; ^13^ YMCA of Greater Toronto, YMCA Canada, Toronto, ON, Canada; ^14^ AIDS Committee of Toronto, Toronto, ON, Canada; ^15^ Rehabilitation Sciences Institute, University of Toronto, Toronto, ON, Canada

**Keywords:** HIV, Goal Attainment Scaling, goal setting, goal planning, online exercise intervention

## Abstract

**Introduction:**

Adults living with HIV may experience various health-related challenges in life. Exercise has been shown to provide numerous benefits. However, the specific goals that individuals aim to achieve through exercise are not well-documented. Our aim was to explore goal setting among adults living with HIV who participated in an online community-based exercise (CBE) intervention.

**Methods:**

We conducted a multi-method, longitudinal study using data from a 12-month online CBE intervention study involving 6-month intervention and follow-up phases. Goal Attainment Scaling was used to quantify the number and types of goals set and achieved at each phase. We analyzed interview data with a subsample to identify experiences with and factors influencing goal setting.

**Results:**

Thirty-two participants initiated the intervention and were included in analyses. The majority were men (69%); median age of 53 years. Participants articulated a median of four goals before and after the intervention, most commonly related to increasing muscle, reducing weight, and improving strength. Approximately 50% of goals were achieved at the end of intervention and follow-up phases. Interview data (*n* = 10) indicated goal setting was influenced by personal health concerns, family, and perceived obligations to research. Most found goal setting personal and helpful, while some experienced challenges.

**Conclusions:**

Adults living with HIV prioritized physical-health-related goals during an online CBE intervention, with diverse experiences influencing their goal-setting process. Findings may inform the design and evaluation of online exercise programs for adults living with HIV.

**Clinical Trial Registration:**

identifier (NCT05006391).

## Introduction

Advances in medical treatment and healthcare have increased the life expectancy of people living with human immunodeficiency virus (HIV) over the past few decades (1–3). In tandem with this achievement, people living with HIV remain at an increased risk of developing other chronic health conditions, such as diabetes mellitus, cancer, and cardiovascular diseases, and experiencing health-related challenges that influence their everyday lives (4–6). These challenges, collectively termed *disability*, may be experienced as episodic in nature and encompass multiple dimensions including physical, cognitive, mental or emotional symptoms, uncertainty about the future, difficulties with day-to-day activities, and challenges to social inclusion (5). Addressing these challenges is important to support the health and well-being of people living with HIV.

Exercise is a well-established rehabilitation strategy shown to provide benefits, including improved physical fitness, neurocognitive health, and everyday functioning, among people living with HIV (7–11). Exercise interventions have traditionally been delivered in gym-based settings, but online platforms have recently emerged as a viable alternative to address individual, environmental, and structural barriers while offering a flexible solution in response to the COVID-19 pandemic (12–14). Collectively, exercise represents a promising accessible strategy to reduce disability among people living with HIV.

To optimize the efficiency and effectiveness of exercise interventions, it is essential to consider *what* people living with HIV aim to achieve through exercise. Goal setting, also known as goal planning, plays a key role in enhancing self autonomy and ensuring that the intervention delivered aligns with individuals' needs, values, and expectations (15–17). With a long history in rehabilitation, it is regarded as a central component through which rehabilitation professionals implement goal setting as a formal approach to establish expected outcomes, personalize interventions, and evaluate progress (16, 18–20). This approach has been examined extensively across various chronic health conditions, such as individuals with multiple sclerosis (21), stroke (17), acquired brain injury (22), neurological impairments (23), and settings, such as geriatric hospitals (24) and outpatient rehabilitation (25). Importantly, a wealth of research has shown that identifying personally meaningful goals not only reduces anxiety but also enhances motivation and treatment adherence (26, 27), and ultimately improves performance and rehabilitation outcomes (17, 28, 29). However, there remains a paucity of research examining goal setting among people living with HIV in the context of online exercise interventions. This lack of knowledge hinders our ability to tailor exercise programs, foster participant engagement, and maximize the potential of such interventions to address the challenges experienced by people living with HIV. To bridge this gap, the objective of this study was to explore goal setting among adults living with HIV who participated in an online community-based exercise intervention. Specifically, we aimed to describe the nature and extent of goal setting and achievement, motivations behind goals, and process of goal setting within an online exercise intervention.

## Materials and methods

### Study design and procedures

We conducted a multi-method, longitudinal, observational study using data from an online community-based exercise (CBE) intervention study (12). The online CBE study was designed to examine the impact and implementation of an online tele-coaching exercise program among adults living with HIV in Toronto, Canada. In partnership with the Toronto YMCA, the study consisted of a 6-month online exercise intervention and a 6-month follow-up. During the intervention phase, participants were instructed to complete 60 min of online, home-based exercise three times per week for 24 weeks. This included a combination of independent exercise, weekly group-based exercise classes, and bi-weekly personal fitness coaching sessions. The group classes and personal coaching sessions were led by certified YMCA fitness trainers. In addition, participants attended monthly online self-management education sessions facilitated by fitness or health professionals. The topics covered in these education sessions included goal setting, dietary strategies, sleep hygiene, yoga and brain health, chronic pain management, and self-coaching. Before the intervention, participants met one-on-one with their YMCA fitness trainer to develop individualized exercise programs involving aerobic, resistance, balance, and flexibility training. Examples of the online multicomponent exercise program are provided in Supplementary Table S1. Prior to program delivery, fitness trainers participated in two knowledge training and exchange workshops to become familiar with the online CBE study and to review the concept of goal setting, importance of, challenges with goal setting, and lessons learned from the prior in-person CBE study (30).

During the follow-up phase, participants retained access to the YMCA online exercise classes and were encouraged to continue independent exercise three times per week. Quantitative assessments were conducted online bi-monthly (Month 0, 2, 4, 6, 8, 10, 12) to evaluate the effectiveness of exercise intervention. We conducted a series of online qualitative interviews at baseline and end of the intervention with a subsample of participants to explore their expectations, experiences, and perceptions of overall study implementation (12). The present study focused on data pertaining to participants' goals collected during the quantitative assessments and qualitative interviews. We provided a schematic overview of the study design and procedures in Supplementary Figure S1. The online CBE study protocol received approval from the research ethics board at the University of Toronto (Protocol # 40410).

### Participant recruitment and eligibility criteria

Participants were recruited via community-based organizations and through the Ontario HIV Treatment Network Cohort Study (OCS) at the Maple Leaf Clinic in Toronto, Canada (31). We specifically targeted adults living with HIV who self-reported challenges exercising in traditional gyms due to geographic, personal, or structural barriers. Participants were screened for the following inclusion criteria: (1) adults aged 18 years or older living with HIV in Toronto; (2) medically stable and deemed safe to participate in exercise based on the self-administered Physical Activity Readiness Questionnaire (32); (3) having access to a smartphone, electronic device, laptop, or desktop computer; (4) having access to Wi-Fi or data internet plan; (5) possessing a web-cam and be willing to use it for group exercise classes, fitness sessions, educational sessions, and online assessments; and (6) self-declared access to sufficient space at home to exercise. The research team provided participants with home exercise and assessment equipment, including resistance bands, a plyo box, and a smart scale.

In accordance with our study protocol, we aimed to recruit 40 participants, with the expectation that 30 would complete the online exercise intervention (12). The sample size was determined based on feasibility considerations informed by our experience in implementing community-based exercise interventions among people living with HIV (33, 34). For the qualitative interviews, we purposively recruited women participants to ensure gender diversity (≥5 cisgender and transgender women) in the interview sample. All participants provided a written informed consent to participate in this study. Details on the CBE study protocol have been previously published (12).

### Data sources and measurements

The primary data source for this study was Goal Attainment Scaling (GAS). GAS has been used in many fields, including rehabilitation, to quantify and monitor progress toward personal goals (20, 35–38). Between October 21 and November 28, 2021, participants completed the baseline GAS to identify their goals for the online CBE study. There was no limit to the number of goals participants could articulate. Each goal was weighted by the participants based on self-perceived importance and difficulty using a 4-point scale (0 = not at all important/difficult; 1 = a little important/difficult; 2 = moderately important/difficult; 3 = very important/difficult). Participants rated their baseline function for each goal using a 2-point scale (−1 = some function; −2 = no function or as bad as they could be). The GAS baseline *T*-score was calculated using the Kiresuk and Sherman formula (39) and follows a normal distribution [mean = 50, standard deviation (SD) = 10]. Higher scores indicate better baseline function with respect to the goals stated. Goals were shared with fitness trainers to tailor exercise programs. At Month 6 (end of intervention phase), we assessed the extent to which participants achieved their goals. Each goal was self-rated by participants on a 5-point scale to reflect the degree of achievement (−2 = got worse; −1 = partially achieved or no change; 0 = achieved as expected; 1 = achieved a little more; 2 = achieved a lot more). Similar to baseline scoring, the GAS attainment *T*-score follows a normal distribution (mean = 50, SD = 10) with a score of 50 indicating goals were achieved as expected. Scores above 50 suggest better than expected achievement, whereas scores below 50 suggest less than expected achievement. We repeated the GAS assessment at Month 6 to assess a new set of goals for the follow-up phase and evaluated goal attainment at Month 12. GAS is considered a reliable instrument and is commonly used for measuring goals in rehabilitation settings (15, 40). All GAS assessments were conducted online via Zoom by two health professionals (physiotherapists) from the research team, both of whom had prior experience with GAS and followed the standardized administration guide to ensure consistency.

We conducted qualitative interviews with a subsample of participants to explore the process of goal setting in the online CBE study. During the Month 0 interviews, participants described the nature and types of goals they aimed to achieve and explained their reasons for setting these goals. At Month 6, participants articulated their experiences with the process of setting goals in this study. In general, interviews were conducted after the GAS assessments, and the goals discussed during the interviews were not limited to those stated in GAS. A research team member with training in HIV and qualitative research conducted the one-on-one interviews using a semi-structured interview guide via Zoom. All interviews were audio-recorded and transcribed verbatim.

Information on participant characteristics including age, gender, race and ethnicity, educational attainment, living status, employment status, year of HIV diagnosis, use of antiretroviral medications, and number of chronic conditions in addition to living with HIV was collected at Month 0 via a self-reported electronic-administered questionnaire.

### Data analysis

We reported descriptive statistics to summarize participant characteristics at baseline. Median [interquartile range (IQR)] was reported for continuous variables, and frequency (%) was presented for categorical variables. We conducted a word frequency analysis on the text collected from the GAS assessments to identify the most frequently stated goals. The analysis was performed in R software (version 4.3.1) using the wordcloud package (41). Additionally, we counted the number of goals set and achieved at each assessment and calculated the corresponding GAS *T*-scores. Goal achievement rate was calculated as the total number of goals achieved divided by the total number of goals set, with possible scores ranging from 0% to 100%. For the interview data, we used a qualitative descriptive approach and first triangulated goals stated in both GAS and interviews to calculate the agreement rate (42, 43). We reviewed Month 0 interview transcripts in detail to identify participants' motivations for setting goals and reviewed Month 6 transcripts to characterize their experiences with the process of goal setting in the online CBE study.

## Results

Of the 33 enrolled participants, 32 completed the GAS assessment at Month 0 and initiated the online exercise intervention phase. Among them, 22 (69%) completed the intervention and GAS at Month 6. The median number of bi-weekly personal fitness coaching sessions attended was 11 out of 13 total sessions (IQR: 6–12). Of the participants who did not complete the exercise intervention (*n* = 10), the reported reasons included having a busy schedule (*n* = 4), unknown reasons (*n* = 4), episodic health issues (*n* = 1), and dissatisfaction with the study (*n* = 1). At Month 12, 18 participants remained in the study, and 17 of them (53% of those who initiated the intervention) completed the final GAS assessment. Reasons for non-completion during the follow-up phase included lost to follow-up (*n* = 3) and health issues (*n* = 1).

Table 1 summarizes the characteristics of participants at baseline. The majority were men (69%), with a median age of 53 years (IQR: 44–59). Most participants reported a median of three concurrent health conditions in addition to living with HIV (IQR: 1–6).

**Table 1:** Baseline characteristics of study participants and subsample who participated in an interview.

Participant characteristics	All participants (Quantitative sample, *n* = 32)	Interview participants (Qualitative subsample, *n* = 10)
	Median (Interquartile range)/*n* (%)	
Age (years)	53 (44, 59)	55 (47, 61)
Gender (*n*)		
Men (Cis-man)	22 (69)	5 (50)
Women (cis-woman)	9 (28)	5 (50)
Non-binary	1 (3)	0
Race/Ethnicity (*n*)		
White	14 (44)	1 (10)
Black or African	8 (25)	5 (50)
South Asian	8 (25)	3 (30)
Latin American, Hispanic, or Latino	2 (6)	1 (10)
Education (*n*)		
Secondary school or below	5 (16)	0
Trade/technical training or college	12 (38)	5 (50)
University education or higher	13 (41)	5 (50)
Employment status (*n*)		
Employed (full time or part time)	15 (47)	5 (50)
Student, retired, or volunteering	8 (25)	3 (30)
Unemployed or on disability	8 (25)	2 (20)
Other	1 (3)	0
Living alone (*n*)	12 (38)	4 (40)
Year of HIV diagnosis	2002 (1992, 2010)	2004 (1993, 2006)
Currently taking antiretrovirals (*n*)	32 (100)	10 (100)
Number of comorbidities (count)	3 (1, 6)	3 (1, 7)

All participants who initiated the online exercise intervention were instructed to complete the quantitative assessments (*n* = 32). A subsample of participants (*n* = 10) was asked to participate in qualitative interviews to provide insights into their experiences in the study.

**Figure 1:**
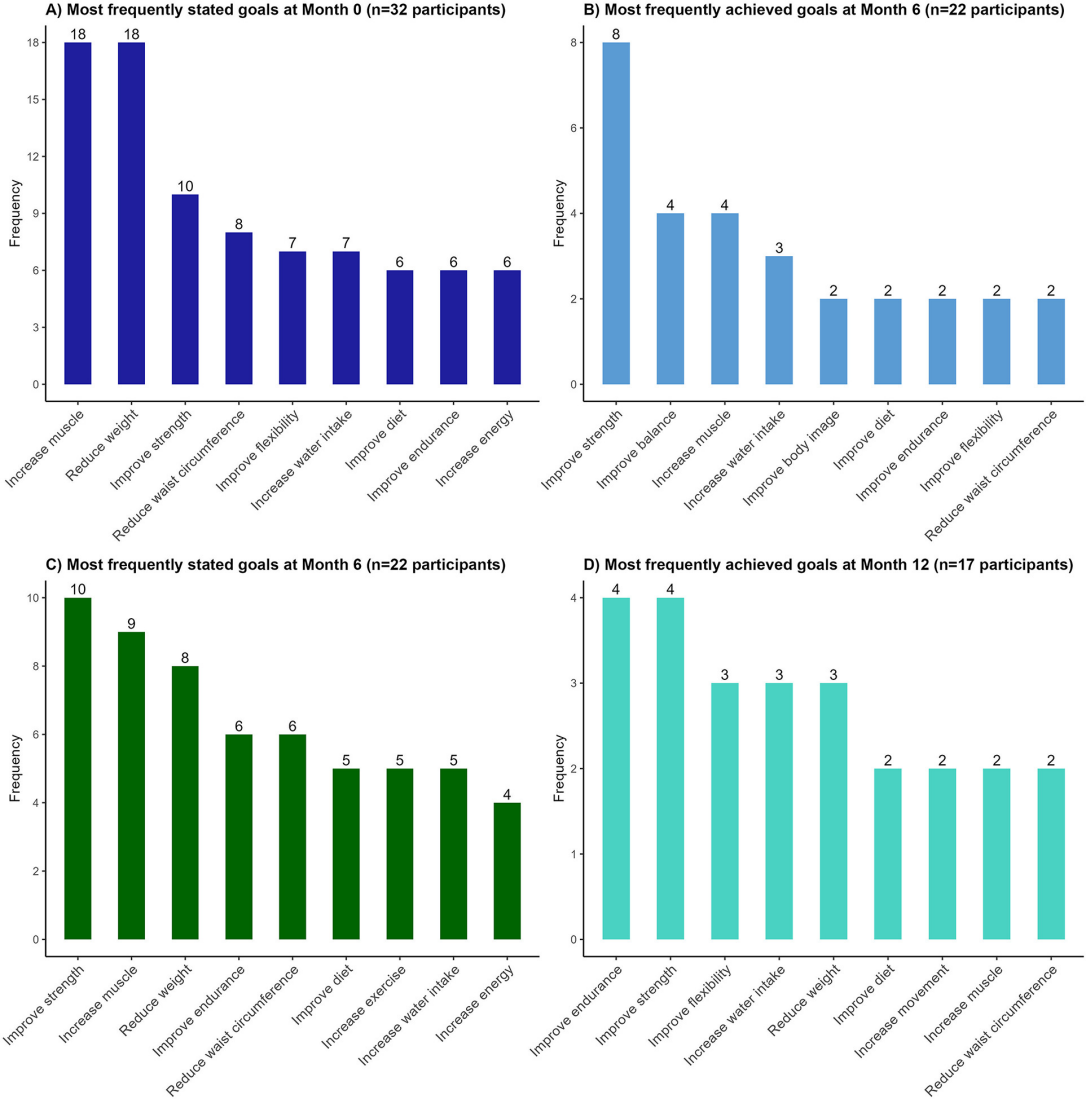
Most frequently stated and achieved goals at each evaluation time point of the online community-based exercise (CBE) intervention study. We used Goal Attainment Scaling (GAS) to quantify the number and types of goals set at Month 0 (start of intervention; dark blue; panel **A**), and then measured how many of these goals were achieved at Month 6 (end of intervention; light blue; panel **B**). We repeated this process to quantify a new set of goals set at Month 6 (start of follow-up; dark green; panel **C**) and assessed how many were achieved at Month 12 (end of follow-up; light blue; panel **D**).

Table 2 presents the number of goals set and achieved at each assessment point. At Month 0 (prior to online exercise intervention), participants articulated a median of four goals (IQR: 3–4), with an average GAS baseline *T*-score of 35.8 ± 1.9. As illustrated in Figure 1, the most frequently stated goals included increasing muscle (*n* = 18), reducing weight (*n* = 18), and improving strength (*n* = 10). By the end of the exercise intervention (Month 6), participants achieved a median of two goals (IQR: 1–3; achievement rate: 46%). The most frequently achieved goals were improving strength (*n* = 8), improving balance (*n* = 4), and increasing muscle (*n* = 4). Additionally, 18 of the 22 participants (82%) achieved at least one of their stated goals at Month 6. The average GAS attainment *T*-score was 47.6 ± 9.6.

**Table 2:** Goal setting and goal achievement at each assessment of the online community-based exercise (CBE) study.

	Goal setting (*n* = 32)	Goal achievement (*n* = 22)	Goal setting (*n* = 22)	Goal achievement (*n* = 17)
	Median (Interquartile range)/Mean ± standard deviation			
Time of assessment	Month 0	Month 6	Month 6	Month 12
# Goals set	4 (3, 4)	—	4 (3, 5)	—
# Goals achieved^a^	—	2 (1, 3)	—	2 (1, 3)
Achievement rate (%)^b^	—	45.9 ± 32.9	—	47.4 ± 35.6
GAS *T*-score	35.8 ± 1.9	47.6 ± 9.6	33.3 ± 5.4	49.8 ± 10.4

^a^ The number of goals achieved was assessed at the next evaluation timepoint (e.g., goals set at Month 0 were evaluated at Month 6, and goals set at Month 6 were evaluated at Month 12).

^b^ The achievement rate was calculated as the total number of goals achieved divided by the total number of goals set.

At Month 6, participants who remained in the study set a new median of four goals for the follow-up phase (IQR: 3–5). The most frequently stated goals were improving strength (*n* = 10), increasing muscle (*n* = 9), and reducing weight (*n* = 8). At the end of the follow-up phase (Month 12), participants achieved a median of two goals (IQR: 1–3; achievement rate: 47%). The most frequently achieved goals were improving endurance (*n* = 4), strength (*n* = 3), flexibility (*n* = 3), water intake (*n* = 3), and reducing weight (*n* = 3). Fifteen of the 17 participants (88%) achieved at least one of their goals at Month 12. The average GAS attainment *T*-score at Month 12 was 49.8 ± 10.4.

A total of 10 participants took part in an interview at Month 0. The baseline characteristics of this subsample are similar to those of the full sample and are presented in Table 1. Among participants interviewed at Month 0, approximately half of the goals stated in GAS were also mentioned in their interviews (Supplementary Table S2). We identified three distinct factors that influenced goal setting in this study: (i) *personal health*; (ii) *family members*; and (iii) *perceived obligations*.

### Setting goals for personal health

Four participants indicated that they set goals due to concerns about their health and challenges experienced in daily activities. For example, one participant stated:

I'm already overweight and because I'm overweight, then I have… sometimes I have a problem with my back, you know the normal small little things you know. So I need to lose that so that I'll be more functional. (P5, woman, 40 years old)

Similarly, another participant who expressed a desire to improve flexibility emphasized how stiffness and lack of movement had begun to impact daily life:

From daily activities that I perform there are times when obviously you start to notice the aches and pains of time that you haven't been either using something or certainly not stimulating the muscle in the right way. So reaching, bending, those sorts of things become more and more difficult over time. (P9, man, ≥50 years)

Another participant aimed to improve lower extremity strength given that weakness in the legs had made everyday activities more difficult:

When I squat to sit down on the toilet or even to squat down to get up, I've got to push up with my arms. My legs are not strong to get me up. I want to strengthen my leg muscles. (P10, woman, 58 years old)

### Setting goals for family members

In addition to personal health concerns, two participants expressed that their goals were influenced by close family members. One participant described how her goal setting reflected the desire to remain healthy for her children:

I think just wanting to be healthy to watch these little kids of mine grow up. I mean he's 8 and my daughter is 12. My mom had four strokes before she passed away. It's something that I worry about. (P1, woman, <50 years)

In parallel, another participant shared how encouragement from his child influenced his decision to formulate health-related goals:

Because my son nags at me all the time. My son nags at me “you should take care of yourself, you’re getting older, you need to take care of yourself and if you don't you’ll die earlier blah blah blah. So you need to live a longer life, a healthier life. If not you’ll grow old and you’ll become sickly and who's going to take care of you.” And on and on and on and he's right actually. I agree with him. (P6, man, ≥50 years)

### Setting goals due to perceived obligations

It is worth noting that one participant expressed that his reason for setting goals was primarily to comply with the research protocol rather than personal motivation:

I've got to say off the top of my head zero thought because let's clarify what a goal would be because I mean in the most general sense my goal is to comply with your minimum standard of compliance. (P4, man, ≥50 years)

The same participant elaborated on why he had rarely focused on setting personal goals:

When we talk about goals I haven't given it a lot of thought because there have been patterns in my life where I always put other needs ahead of my own, other people's needs ahead of my own. (P4, man, ≥50 years)

### Experiences with setting goals in the online CBE study

Nine participants took part in an interview at Month 6 (eight of whom also completed the interview at Month 0). Of these participants, seven (80%) possessed a positive attitude toward setting goals in this study. For instance, one participant expressed that goal setting was helpful because it aligned with personal preferences and expectations:

Yeah, it is helpful because the goal when I set the goal, it was based on my own issue. So I want to reduce my weight. I want to keep my exercise as a habit. So those are all what I dream for. So that is all good for me. Yeah. (P7, woman, ≥50 years)

This perspective was echoed by another participant, who described how goal setting helped make objectives more concrete and specific while also fostering a sense of accountability:

Yes, absolutely because you put into words what goals are. It makes you think about your goals and the steps to get there and in order for you to evaluate how those steps are going. So you've made concrete a lot of things that just could be vague and nebulous. (P4, man, ≥50 years)

However, two participants (22%) expressed challenges with goal setting in the online CBE study. Specifically, one participant stated:

So I can't… I'm not that type of thinker. It's got to be in that moment because a lot of this is all goal setting and I'm terrible at it. In the beginning I was like sure, that sounds like a great goal, we'll do that and I know I wasn't going to do it because it wasn't my goal. I’m just horrible with goals. (P2, woman, <50 years)

The other participant expressed mixed feelings about setting goals and highlighted the concern that setting specific goals can be daunting:

But the way it was… I was… I saw it… I had to be very specific with my goals and I think that is a very demotivating thing, definitely. Because right now because it's very specific I feel like a failure. I feel like this whole exercise… whereas if I had just kept to what my original goal was for joining this thing was to get healthy, it's been a success. It's perspective, how you look at it right. (P6, man, ≥50 years)

## Discussion

We explored goal setting among adults living with HIV who participated in an online CBE intervention. Participants set a median of four goals, primarily related to increasing muscle, reducing body weight, and improving strength. Factors influencing goal setting were multifaceted, ranging from personal health concerns to encouragement from family members and perceived obligations as research participants. Importantly, the majority of participants expressed that goal setting was helpful while some faced challenges with this process.

To our knowledge, this study is the first to investigate goal setting among adults living with HIV in an online exercise intervention. Although direct comparisons with other studies are limited due to differences in settings and instruments used, our findings are consistent with previous research on exercise interventions for people living with HIV. For instance, Montgomery et al. (44) reported that people living with HIV who participated in an in-person community-based exercise program reported goals such as gaining strength and muscle mass, managing body weight, reducing pain, and increasing energy. Similarly, Homayouni et al. (45) found that people living with HIV and complex multimorbidity often set goals around managing concurrent health conditions, remaining mobile and functionally independent, and enhancing physical fitness while participating in a physiotherapist-led exercise program. More recently, Iriarte and colleagues (46) showed that improving body image and being in shape were key drivers for older adults living with HIV to participate in exercise trials. It is worth noting that the goals identified in this study mainly focused on physical health and were consistent with the benefits of exercise documented in the literature. For example, prior systematic reviews and meta-analyses have shown that aerobic and/or resistance exercise can improve strength, body composition, and cardiorespiratory fitness among adults living with HIV (47, 48). This alignment has important implications. First, the congruence suggests that participants were aware of the benefits of exercise and set goals directly reflected the nature and intended outcomes of the online CBE intervention. Second, this finding may reflect a specific challenge, namely the impact of lipodystrophy, experienced by people living with HIV (49). Lipodystrophy, characterized by fat redistribution, is a known side effect of long-term use of highly active antiretroviral therapy (HAART). Lipodystrophy is associated with body image dissatisfaction, lower self-esteem, and depression, which can lead to feelings of loss of control and, in some cases, decreased adherence to HAART (50–52). Given these challenges, it is possible that participants in this study prioritized goals related to body composition and physical fitness as a way to mitigate the influence of lipodystrophy, improve body image, and regain a sense of control over their health. Overall, these findings underscore the importance of understanding the most commonly stated goals of adults living with HIV and help inform the design, implementation, and evaluation of exercise interventions tailored to this population. Rehabilitation professionals, for instance, could use these insights to personalize exercise programs and offer targeted support to assist participants in achieving their desired outcomes.

In conjunction with goal setting, we applied GAS to explore goal achievement during and after the online CBE intervention. It is important to clarify that determining the overall effectiveness of the intervention was beyond the scope of this study. However, GAS offers insights into participants' self-appraisal and may complement traditional outcome evaluations. We observed that the mean GAS *T*-scores increased from 35.8 to 47.6 during the intervention phase and from 33.3 to 49.8 during the follow-up phase. The extent of change is comparable to those reported in studies involving rehabilitation interventions among people with neurological conditions such as stroke or Parkinson's disease (53–55). Nevertheless, interpretation and practical use of GAS *T*-scores require further investigation as debate continues in this area (56–58). To enhance interpretability, we reported goal achievement rates and found that approximately 50% of stated goals were achieved at both timepoints, and the most frequently achieved goals pertained to improvement in physical fitness (e.g., strength, balance, endurance, and flexibility). The nature and task-specificity of the online CBE intervention may help to explain why improvements in these goals were observed. It is important to note that goal attainment was self-reported by participants, and that they may have achieved other goals or attained benefits that were not articulated during the GAS assessment. Thus, the goal achievement rates alone may not fully reflect the overall value or impact of the intervention. Given that over 80% of participants achieved at least one of their stated goals, our findings suggest that online coaching and exercise may serve as a promising avenue to help adults living with HIV work toward their valued physical health goals.

Our study employed a multi-method approach and incorporated qualitative interviews to gain a deeper understanding of the underlying motivations for goal setting. We found that goal setting was influenced by a variety of factors, including personal health concerns, encouragement from close family members, and the sense of obligation as research participants. These findings are congruent with previous studies examining facilitators and barriers to exercise among people living with HIV. Regarding personal health concerns, for instance, a qualitative study of women living with HIV by Sahel-Gozin et al. (59) identified the aspiration to stay healthy as a key motivator for engaging in exercise. With respect to close relationships, prior research also revealed that social environments, including support or pressure from friends and family members, can influence whether older adults living with HIV participate in exercise interventions (46). Notably, one participant in our study indicated that they rarely set personal goals and only did so out of a perceived obligation to comply with the study. Throughout the interview, this participant appeared highly accommodating, expressing a willingness to follow whatever the research team requested. This highlights the importance of providing clear, participant-centred guidance when introducing goal setting in the field (60). It should be made explicit that the purpose of goal setting is to support the individual's own priorities, not merely to meet the research team's expectations. Taken together, our study underscores the complexity of goal setting among adults living with HIV and highlights the influence of individual, inter-personal, and social factors on this process. Future research could investigate the interplay of these multilevel factors to optimize goal setting strategies among this population.

Overall, the majority of participants possessed a positive attitude toward goal setting and found it personal, relevant, and helpful in this online CBE study. Our findings emphasize the role of goal setting in implementation science and align with rehabilitation research and practice, where goal setting is considered an effective strategy to empower participants, support the uptake of evidence-based interventions, and promote adherence to exercise programs (28, 61–63). However, some participants expressed difficulties and challenges with goal setting, underscoring that goal setting is a skill that does not come easily to everyone, particularly for individuals living with chronic health conditions who may experience complex life situations and unpredictable periods of wellness and illness (5, 6, 23, 64). Our findings emphasize the need to acknowledge individual differences in goal setting and to provide resources and guidance to support adults living with HIV in this process. Applying rigid or prescriptive goal-setting frameworks that do not consider the needs, circumstances, and capacities of participants may negatively influence motivation and engagement in exercise. There is no one-size-fits-all solution for setting or attaining personal goals. To turn these insights into practice, it is important that future research and clinical applications embrace a person-centred and flexible approach to goal setting, moving beyond the notion that goals must always be SMART (specific, measurable, achievable, realistic/relevant, and time-bound) (25).

Our study is the first known to incorporate both quantitative GAS assessments and qualitative interviews to evaluate goal setting and achievement among adults living with HIV across multiple time points. GAS provided valuable insights into the total number and types of goals participants aimed to pursue, while interviews offered a richer understanding of participants' experiences with goal setting. Despite these strengths, our study has limitations. First, the number of participants who completed the 6-month online exercise intervention (*n* = 22) was below the planned sample size (*n* = 30), with half of the participants completing the 6-month follow-up. This reflects the common challenges in longitudinal research involving multiple components and extended study period. While we collected information on reasons for dropout, attrition bias cannot be ruled out and should be taken into consideration when interpreting the findings. Second, only a subsample of the participants was invited for interviews, which limited our ability to capture the full range of experiences related to goal setting in this study. Third, our inclusion criteria may have introduced positive selection, as participants were primarily adults living with HIV in urban settings who received a high level of education and had access to internet technologies. The transferability of our findings to the broader population of people living with HIV requires further verification. Notably, this study was conducted during the COVID-19 pandemic, which may have influenced the types of goals set and introduced potential period effects that should be considered in interpretation. Lastly, as weight reduction was among the most frequently stated goals, assessing participants' eating patterns would have provided valuable insights into how other lifestyle factors may have supported or hindered progress toward this goal.

## Conclusion

In conclusion, this study explored goal setting and achievement among adults living with HIV involved in an online exercise intervention. We identified that participants set goals primarily related to physical health and uncovered factors influencing goal setting at the individual, inter-personal, and social levels. Findings also indicate that online exercise has the potential to help adults living with HIV attain some of their desired goals. These findings contribute to a more nuanced understanding of goal setting and achievement and may help inform the design, customization, modification, and evaluation of online exercise programs to better meet the needs and preferences of adults living with HIV and potentially other chronic health conditions. Future research could benefit from further exploring the alignment between participant goals and fitness trainer perspectives to strengthen rapport, support collaborative goal setting, and enhance the overall success of online exercise interventions.

## Data Availability

The data analyzed in this study is subject to the following licenses/restrictions: Data cannot be shared publicly as participants did not consent to providing public access to their data in the consent process. All relevant data supporting the findings are within the manuscript and its Supporting Information files. For more information regarding data availability for this study, individuals may contact the Research Ethics Board, University of Toronto. Requests to access these datasets should be directed to ethics.review@utoronto.ca.
