# Supplementary material for: Goals in motion: exploring goal setting among adults living with HIV who participated in an online community-based exercise intervention
Source: Front Rehabil Sci. 2025 Jul 31;6:1644139. doi: 10.3389/fresc.2025.1644139 (PMC12350490; doi:10.3389/fresc.2025.1644139)
Supplement: Supplementary file 1 [file Table1.docx]

**Supplementary Table 1.** Example of the online, multicomponent exercise program

|  | **Warm Up** | **Aerobic** | **Balance** | **Strength** | **Flexibility** | **Cool Down** |
| --- | --- | --- | --- | --- | --- | --- |
| Frequency | Before each exercise session | Minimum 3 days/week | After warm up/aerobic portion of each exercise session, also integrated into some strength exercises | Minimum 3 days/week | Daily | After each exercise session |
| Intensity | Light | Moderate initially, vigorous can be reached with gradual progression | 3 holds for static balance exercise, tailored instructions for dynamic balance exercise | Light to moderate  (8 – 15 repetitions) | Stretch to a point of tension, but not pain | Light |
| Time  (60 min total for each session) | 5 – 10 minutes | 20 – 30 minutes of continuous activity (can be broken into bouts of 5 to 10 minutes) | 30 – 60 seconds per static balance hold | 8 – 10 exercises within 45 to 50 minutes | 30 seconds per stretch | 5 to 10 minutes |
| Type | Walking, light aerobic, dynamic and static stretching | Any enjoyable activity that increases heart rate | Can be static (e.g., one leg stance) or dynamic (e.g., walking heel to toe) | Resistance bands and body weight | Static stretching | Walking, light aerobic, dynamic and static stretching |

*Note*: This table provides an overview of the online exercise program structure. Actual exercise programs were tailored to each participant’s goals, abilities, and preferences through one-on-one sessions with certified YMCA fitness trainers.

**Supplementary Table 2.** Summary of goals reported in the Goal Attainment Scaling (GAS) and qualitative interview at Month 0

| **P** | **Goals stated in Goal Attainment Scaling (GAS)** | **Goals stated in interview** | **Agreement** |
| --- | --- | --- | --- |
| 1 | 1. Lose weight 2. Use exercise to gain more energy 3. Utilize recreation space near home 4. Maintain daily walking throughout the winter 5. Improve blood pressure through exercise 6. Improve meal preparation habits, portion control, and adherence to DASH diet & intermittent fasting recommended by doctor | 1. Get the energy (tackle anything) 2. Lose weight | 2/6 = 33% |
| 2 | 1. Improve balance 2. Improve flexibility 3. Increase physical activity outside of work hours 4. Maintain 4 days of working out each week even if friends bail 5. Increase water intake | 1. Improved strength 2. Lower blood pressure 3. Lower cholesterol 4. Build the habit of exercise | 1/5 = 20% |
| 3 | 1. Improve sleep 2. Lose weight 3. Decrease waist circumference 4. Decrease pain | 1. Lose weight 2. Live longer 3. With less pain | 2/4 = 50% |
| 4 | 1. Have the energy to do something that is fun 2. Have more muscle mass 3. Increase hand grip strength | 1. Feel fit 2. Get more energy | 2/3 = 67% |
| 5 | 1. Lose weight 2. Be more flexible 3. Have at least 8 hours of sleep per night 4. More water intake and space out water throughout day instead of having one large bottle once during the day 5. Eat healthier | 1. Get the whole exercise in system (enjoy going to the gym and workout) 2. Be healthy again, monitor activities, diet, and exercise 3. Lose weight 4. Make exercise a habit | 2/5 = 40% |
| 6 | 1. Lose weight 2. Gain some biceps 3. Bigger chest/pecs 4. Lose belly 5. Tighten buttocks 6. Broader and stronger shoulders | 1. Lose weight | 1/6 = 18% |
| 7 | 1. Reduce weight 2. Gain more physical energy 3. Make exercise a regular habit | 1. Motivate self to do the exercise (making exercise as a habit) 2. Reduce weight 3. Keep body a little active so that don’t feel tired | 3/3 = 100% |
| 8 | 1. Increase muscle, return to “self-ideal body” 2. Meditation 3. Improve cardio | 1. An elevation in physicality 2. Build up the muscles more in certain areas 3. Reconnection to previous state or goals (keep on track) | 2/3 = 67% |
| 9 | 1. Improve personal competitive swim times 2. Increase strength/build muscle 3. Improve balance (in and out of the water) 4. Increase movement and efficiency (in and out of the water) 5. Increase flexibility 6. Increase mindful focus on movement | 1. Have stable muscle mass 2. Have bigger arms 3. Improve strength 4. Improve flexibility | 2/6 = 33% |
| 10 | 1. Lose 25lbs before turning 60 years old 2. More strength in arms 3. More strength in legs | 1. Lose 25lbs before turning 60 years old 2. Be fitter (have a fitter bum) 3. Have stronger legs 4. Have stronger arms | 3/3 = 100% |

*Note:* The agreement rate was calculated as the proportion of goals stated in the quantitative assessment (GAS) that were also mentioned during the qualitative interview. This report is based on a subset of 10 participants who were invited to participate in the qualitative interviews

**Supplementary Figure 1.** Schematic overview of the study design and procedures of the online community-based exercise (CBE) intervention study


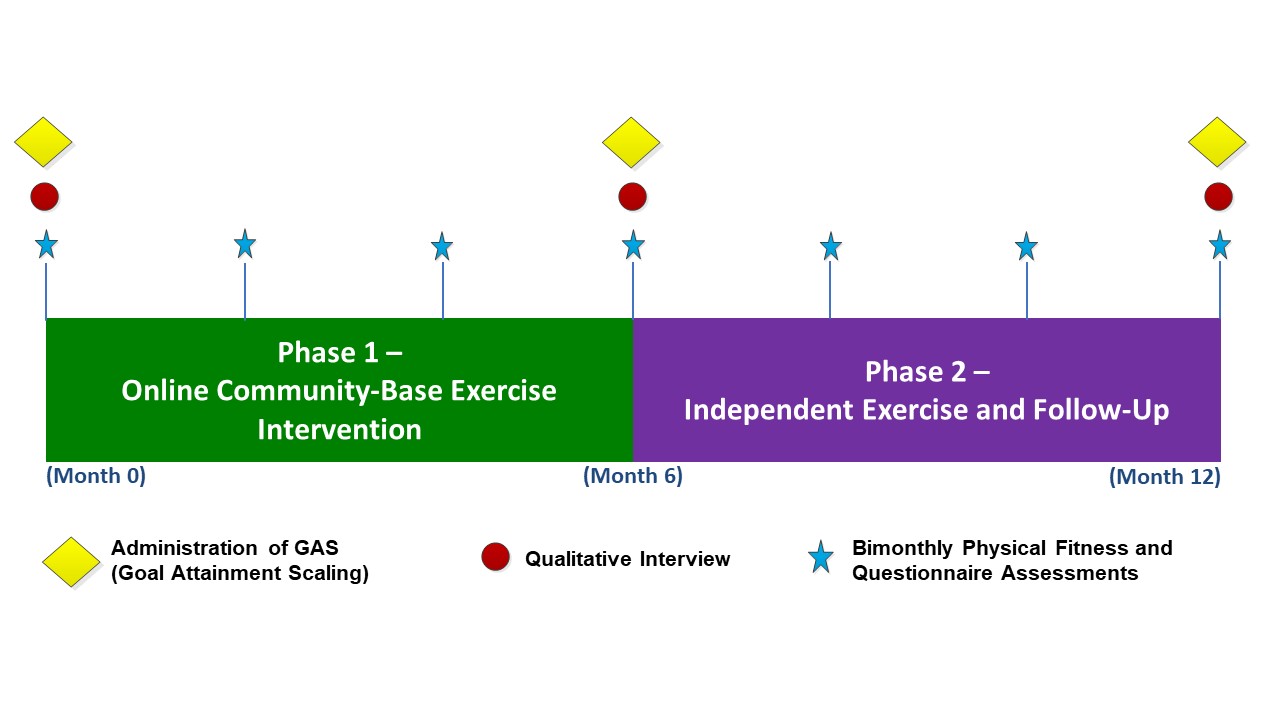


*Note*: The online CBE intervention study consisted of a 6-month intervention phase followed by a 6-month follow-up phase. Participant screening, informed consent, and recruitment occurred prior to the start of the intervention (i.e., before Month 0).
